# Prevalence of Axillary Artery Variants and Their Clinical Significance: A Scoping Review

**DOI:** 10.7759/cureus.47809

**Published:** 2023-10-27

**Authors:** Leonidas Brilakis, George Tsakotos, Panagis M Lykoudis, Maria Piagkou, Theodore Troupis

**Affiliations:** 1 Department of Anatomy, School of Medicine, Faculty of Health Sciences, National and Kapodistrian University of Athens, Athens, GRC; 2 Division of Surgery and Interventional Science, University College London, London, GBR

**Keywords:** upper limb, surgery, anatomy, variation, axillary artery

## Abstract

Axillary artery (AA) variants occurred quite commonly, presenting clinical implications. A literature search yielded 523 results from which 13 parameters were extracted. Some of the AA variants found were the fusion of two or more branches into common trunks, like the fusion of anterior and posterior circumflex humeral arteries. Moreover, several branches were found to emerge from different points than expected, like the lateral thoracic artery’s origin from the subscapular artery instead of the second part of the AA. The importance of the knowledge of the AA variations in clinical practice is undeniable and very useful when planning interventional procedures, as in the case of AA aneurysm treatment or in cases of fracture of the surgical neck of the humerus. The heterogeneity of data limited the possibility of a quantitative summary of data. Therefore, a more systemic study of AA variants based on the origin, course, and branching pattern is suggested. The aim of the current review is to summarize current data literature regarding the AA typical anatomy and its variants, with a focus on their prevalence and possible clinical implications.

## Introduction and background

Variants of the axillary artery (AA) branching pattern are considered quite common. The AA is the continuation of the subclavian artery, beginning at the lateral border of the first rib and continuing until the inferior border of the teres major muscle, where it serves as the origin of the brachial artery (BA). The AA is divided by the pectoralis minor muscle (Pm) into three parts: the first part is located medial to the Pm, the second one, posterior to the Pm, and the third part, lateral to the Pm [1]. It is well-known that the AA typical branching refers to one branch, the superior thoracic artery (STA), arising from the first part; two branches from the second part, the thoracoacromial artery (TAA) and the lateral thoracic artery (LTA); and three branches from the third part, the subscapular artery (SSA) and the anterior and posterior circumflex humeral arteries (ACHA and PCHA) [2].

There are several types of variants. The AA branching pattern may highly vary from the typical anatomy. According to Thiele et al. [3], the AA typical branching pattern was found in less than 20% of the study sample. The number of branches also varies, with DeGaris et al. [4] reporting a median number of eight branches, with a range from five to 11 branches. AA length also varies with a median length of 10.15 cm [5]. The variants of the AA branching pattern are very important for cardiac and thoracic surgeons, as the AA branches are used for coronary artery bypass [1]. Moreover, it is very useful for preventing bleeding intraoperatively in cases of chronic shoulder dislocation procedures by orthopedic surgeons [6].

The current scoping review summarizes current data literature, regarding different variants and their prevalence with a particular focus on their clinical implications.

## Review

Literature search

**Figure 1:**
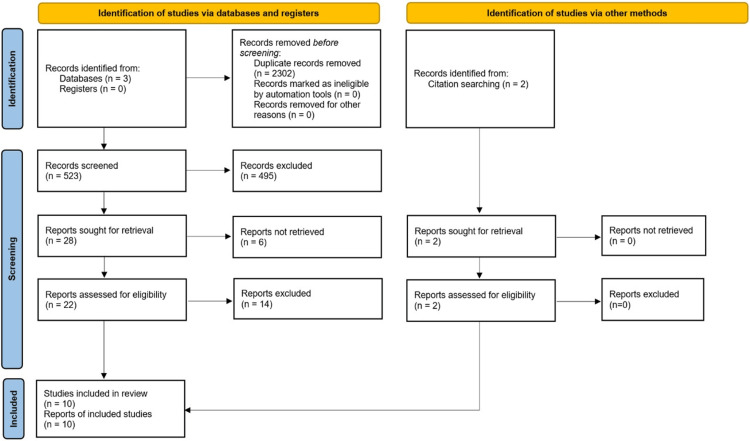
The flowchart of the review. The methodology steps that were followed to include and exclude the identified studies.

The search words for the data literature review were arranged in three groups: the first group consisted of the keywords “axilla,” “axillary,” “armpit,” and “underarm”; the second, of the keywords “artery,” “arterial,” “vascular,” and “vessel”; and the third group consisted of the keywords “variations,” “variation,” “variability,” “aberrant,” and “unconventional.” The search terms were created by combining one word from each group to create all the possible combinations. The search terms that were created were 80, and they were sought in titles and/or abstracts of English language papers appearing in any of the following three databases: PubMed (National Library of Medicine), Scopus, and Web of Science. Regarding Web of Science, the authors of this study performed the search with the terms ((axillary OR axilla OR armpit OR underarm) AND (artery OR arterial OR vascular OR vessel) AND (variations OR variation OR variability OR aberrant OR unconventional)) in two fields separately. The first field was the title, and the second field was the abstract as there is no option for a combined search at once. References from relevant papers were also screened to identify possible additional articles. The last search was performed on May 25, 2023. Figure 1 depicts the flow of articles' screening and selection progress.

Inclusion criteria

Single case studies and single case reports were excluded. There was no limitation as far as the year of publication was concerned. Only studies that included humans were processed. 

Data collection

Thirteen parameters were extracted and recorded. The first four concerned the name of the first author(s), the year of publication, the country of origin or the sampled population, and the sample size of the study (n=number of investigated axillae). The fifth parameter concerned the gender of studied individuals, expressed as the number and percentage of males. The sixth parameter concerned the mean length in cm that each study reported. The seventh parameter included the number and percentage of specimens where typical anatomy was detected. The eighth parameter concerned the range of branches that were reported. The ninth parameter concerned the number and percentage of the origin of LTA from the SSA. The tenth parameter concerned the incidence of the common trunks’ (CT) formation from the ACHA, the PCHA, and the SSA, as absolute numbers and percentages. The eleventh parameter concerned the incidence of CT formation from the ACHA and PCHA, reported as absolute numbers and percentages. The twelfth parameter concerned the incidence of CT formation from the PCHA, the SSA, and the LTA, reported as absolute numbers and percentages. Finally, the thirteenth parameter concerned the number and percentage of specimens where the PCHA originated from the SSA.

Results

Ten studies contributed to data extraction, and Table 1 summarizes key information. The included studies examined cadaveric specimens, and the sample's size ranged from 24 limbs [7] to 840 limbs [8]. The sampled populations included cadavers from a wide geographical distribution such as Korea, Asia, Central Europe, and South Africa. The cadavers were both males and females with the smaller percentage of male cadavers, 62% [8], while the highest percentage of male cadavers was 85% in Astik et al.'s study [9]. The incidence of typical anatomy was found to widely vary between different studies, with Konarik et al. reporting typical anatomy to be as low as 23% [10]. Conversely, Olinger et al. reported typical anatomy to be as high as 78.3% [11].

**Table 1:** The prevalence of the axillary artery variants among studies in different populations and geographic origins. ‡ median ¶ along with PBA ✓ without LTA ⨑ referring to LTA/SSA typical anatomy LTA: Lateral Thoracic Artery SSA: Subscapular Artery ACHA: Anterior Circumflex Humeral Artery PCHA: Posterior Circumflex Humeral Artery PBA: Profunda Brachii Artery CT: common trunk N/D : no data –/ Country name: the study was conducted in the specified country (e.g., America, Brazil), but the exact origin or characteristics of the studied population within that country are not provided.

Author(s)	Year	Sampled population / country	sample size (n)	Males (n,%)	Length (cm,mean)	Typical anatomy (n,%)	Number of branches (range)	LTA from SSA (n,%)	CT of ACHA and PCHA and SSA (n,%)	CT ACHA-PCHA (n,%)	CT of PCHA-SSA-LTA (n,%)	PCHA from SSA (n,%)
Yang et al. [12]	2021	Korean	59	N/D	11.057 ‡	15 (25.40%)	3–6		2 (3.38%)	19 (32.00%)	1 (1.69%)	
Astik and Dave [9]	2012	Asian	80	34 (85%)	N/D	30 (37.50%)	4–8	16 (20.00%)	8 (10.00%) ¶	12 (15.00%) ¶		
Huelke [13]	1959	79 white, 10 African American	178	67 (75.28%)	N/D			45 (25.00%)		20 (11.20%)		27 (15.20%)
Konarik et al. [10]	2020	Central European	423	N/D	N/D	95 (23.00%)				59 (13.95%)	97 (22.93%) ✓	
Loukas et al. [8]	2013	– / America	840	236 (62.62%)	N/D			32 (3.93%)				
Olinger and Benninger [11]	2010	– / America	166	N/D	N/D	130 (78.30%) ⨑		7 (4.20%)				20 (12.00%)
Fontes et al. [7]	2015	– / Brazil	24	N/D	N/D					2 (8.30%)		13 (54.20%)
Naidoo et al. [14]	2014	– / South Africa	100	N/D	N/D				3 (3.00%)	31 (31.00%)	15 (15.00%) ✓	2 (2.00%)
Ojha et al. [15]	2015	– / India	60	20 (66.66%)	N/D	40 (67.00%)	5–11		8 (13.00%)		8 (13.00%)	
Trotter et al. [16]	1930	– / America	384	155 (80.72%)	N/D	175 (45.57%)		36 (9.37%)		88 (22.91%)		51 (13.28%)

The authors of the current study observed several variants concerning the AA branching pattern. Only one study referred to the AA length and found the AA median length to be 110.57 mm [12]. LTA typically originates from the AA second part. In many studies, LTA was found to originate from the SSA or one of its terminal branches [13]. Both ACHA and PCHA were found to widely vary from the index origin. Naidoo et al. [14] reported CTs that gave origin to ACHA-PCHA and SSA, and they also reported CTs that gave origin to ACHA and PCHA. PCHA was also found to originate from CTs with SSA and LTA [10]. Additionally, PCHA was observed to originate from SSA instead of the AA third part [14]. Even though most studies did not report the number of branches originating from the AA, some did. The range of the number of AA branches varies from three to 11 branches [12,15].

In several studies, there was significant difficulty in reaching a safe conclusion by performing calculations on the presented data, regarding the frequency of the exact occurrence of a single variant. In some studies, it was difficult to summarize the incidence of a variation as there was not an accurate system of data reporting. In some studies, there was the risk of double reporting of a single variant as this specific variant was analyzed from two different perspectives of the two different branches that participated in the variant [1].

Regarding the laterality of each variant, it was quite difficult in most studies to come to a safe conclusion even though some studies reported relevant information. Naidoo et al. [14] reported that the PCHA originated along with the ACHA from a CT in 31% of the studied specimens (31 specimens, 17 were left-sided, and 14 were right-sided). They also reported that the PCHA formed CTs with the SSA in 27% (27 specimens, 14 were right-sided, and 13 were left-sided) [14]. Trotter et al. [16] reported the common origin from the ACHA and PCHA by a CT in 88 upper limbs (26 on the right, 18 on the left side, and 46 on both sides). It is important to emphasize that although the authors reported 90 sides, we considered the 88 specimens, wherein the CTs directly originated from the AA, while the other two arose from the SSA [16].

Most of the studies do not make special mention of the importance of the AA variations in daily clinical practice. Thiele et al. [3] highlighted the usefulness of the knowledge of the most common variations in the assessment of imaging. They additionally mentioned the importance of this type of variation in procedures like axillary coronary bypass shunts. Moreover, they found this knowledge clinically pertinent to trauma-related reconstruction, the application of regional anesthesia to the brachial plexus, fractures of the surgical neck of the humerus, as well as to the management of AA thrombosis [3]. Tiwari et al. reported the significance of specific types of variations during operations, such as in breast augmentation and mastectomy [1]. More specifically, Thiele et al. [3] referred to the great variability of the LTA, which supplies the nipple-areolar complex, and the need for an accurate assessment of the anatomy of this specific vessel. Shantakumar et al. [2] also mentioned that the AA branching pattern variation should be considered while performing a bypass between the AA and subclavian artery in the case of subclavian artery occlusion. Moreover, they underlined the clinical relevance in cases of aneurysm or trauma in AAs that need to be operated on. Additionally, the AA can be successfully punctured for arterial access required during catheterization for cardiopulmonary bypass [2]. Singh et al. [6] mentioned that in the case of chronic shoulder dislocation, the surgeon must be aware of the existence of any variation because of the high risk of bleeding, intraoperatively. It is common for the AA to be injured or even ruptured during the reduction of the shoulder joint dislocation that occurs long after the dislocation itself [6].

The authors of the current scoping review faced difficulties in concluding the variability of the AA branches as a single entity. Although there are many studies on this topic, how the data are presented can often lead to the formation of erroneous conclusions or the omission of information. For instance, it was very challenging to retrieve data about the side of the variations (right or left). The morphometric characteristics of the AA (e.g., the AA length of the studied subjects) were very limited.

Discussion

The authors of the present critical review study found the AA variability to be higher in the third part, with the second part following, while the first part was highly constant, similar to Chakraborty’s and Sarkar’s findings [17]. Loukas et al. [8] described many variants regarding the LTA. Loukas et al. [8] and Huelke [13] found the LTA originating from the SSA, with percentages ranging between 3.93% and 25%. A common variant was the fusion of some arteries, in the form of CTs. For example, ACHA and PCHA often originated by a CT along with the LTA or the SSA [12]. Yang et al. [12] reported ACHA and PCHA originating from a CT in 19 upper limbs, a percentage of 32%. The typical anatomy appeared to significantly vary between different surveys. Konarik et al. [10] found typical anatomy to be only 23%, while Olinger et al. [11] reported typical anatomy as high as 78.3%. Huelke et al. [13] reported the PCHA originating from the SSA in 27 upper limbs, a percentage of 15.2%.

Regarding the variability of each part of the AA [2], some surveys reported that the first part of the AA is quite constant [17,18]. For instance, out of 50 upper limbs, Gaur et al. reported no variants in the first part [18]. Similarly, Chakraborty et al. also found no variability [17]. Ming Tzu, in agreement with the findings of the current study, reported that the proximal two branches of the AA are mostly constant, and the distal branch is the least constant branch, with the middle branches being intermediate regarding their consistency [19]. De Garis et al. [4] also reported the common origin of the ACHA and PCHA by a CT in 102 cases. The present review similarly recorded this pattern frequently. The study of Huelke [13] and Fontes et al. [7] found the LTA originating from the SSA with percentages of 25% and PCHA originating from the SSA with high percentages of 54.2%. Farhan et al. [20] reported the LTA originating from the SSA in 5% and the PCHA originating from the SSA in 11%.

The meticulous knowledge of the AA variants has an educational value as during dissection, the identification of the aberrant branches is fundamental for their maintenance. Additionally, safe surgery in the axillary area predisposes the in-depth knowledge of the AA variant branching pattern. Thus, the present study could provide further guidance for a more systematic approach to the learning of the AA variants, an issue that would be useful in future anatomy textbooks. Several studies provide us with evidence of the clinical significance of the AA variants. Singh et al. [6] found the comprehensive knowledge of the AA variations particularly useful for the treatment of tumors, abscesses, and traumas in the axillary area and for the axillary lymph node dissection in breast cancer. They also found this knowledge useful in cases like reconstructive surgeries for trauma and aneurysms of the AA, which is very common in baseball players [6]. Singh et al. [6] reported that the prevention of bleeding during the reduction of old shoulder dislocation requires an excellent knowledge of the AA variants. Tiwari et al. [1] highlighted the necessity of this knowledge during treating AA thrombosis, using the medial arm skin flap, reconstructing trauma, or AA hematoma, in cases of axillary-coronary bypass shunt and fractured upper humeral end. Moreover, they reported the proposal of the feeling of the AA pulsations to be a crucial landmark during brachial plexus block or subclavian vein puncture [1]. Shantakumar et al. [2], in addition to previous knowledge, reported that AA has been used as the catheterization site for thoracic and aortic procedures, cardiopulmonary bypass, and even for inserting intra-aortic balloon pumps. Moreover, they reported that the above interventions must occur after an imaging examination to ascertain the anatomy of the area [2]. Thiele et al. [3] reported the usefulness of the knowledge of the AA variants for the application of the brachial plexus local anesthesia.

Regarding the quality of the studies included in the current scoping review, the authors would like to address several issues. The laterality of each variant (identification per side) is often omitted or it is incompletely reported. Moreover, the reporting heterogeneity made drawing conclusions and elaborating on the existing studies difficult. Finally, in contrast to the abundance of recorded AA variations, little mention was made of their relevance to daily clinical practice and the necessity of their pre-procedural determination by clinicians. Thus, the lack of large sample descriptive studies of randomized interventional studies and the extensive heterogeneity of the included studies made the systematic approach of the data with their meta-analysis impossible.

## Conclusions

The current scoping review highlights the wide range of the AA typical anatomy, as well as the high morphological variability of the AA distal (third) part. Moreover, it highlights the fusion of some AA branches into CTs. Despite the existing heterogeneity of the AA-reported variants (data on their prevalence, origin, course, and branching pattern) the value of their meticulous knowledge remains quite important. Based on this necessity, future (systematic) studies should be planned, based on a unified protocol, among different populations. Additionally, the impact of laterality and gender dimorphism of a variant must be investigated, as well as the imaging of the variants. Finally, there is a high necessity for planning large clinical studies in patients aiming to investigate the typical and variant anatomy of the AA and correlate the extracted data with the pathologic background.
